# Differentiating low- and high-proliferative soft tissue sarcomas using conventional imaging features and radiomics on MRI

**DOI:** 10.1186/s12885-024-13339-7

**Published:** 2024-12-30

**Authors:** Fabian Schmitz, Hendrik Voigtländer, Dimitrios Strauss, Heinz-Peter Schlemmer, Hans-Ulrich Kauczor, Hyungseok Jang, Sam Sedaghat

**Affiliations:** 1 Department of Diagnostic and Interventional Radiology, University Hospital Heidelberg, Heidelberg, Germany; 2 Division of Radiology, German Cancer Research Center, Heidelberg, Germany; 3 Department of Radiology, University of California Davis, Davis, CA USA

**Keywords:** Soft tissue sarcoma, Ki-67, ADC, Contrast dynamics, Radiomics

## Abstract

**Background:**

Soft-tissue sarcomas are rare tumors of the soft tissue. Recent diagnostic studies mainly dealt with conventional image analysis and included only a few cases. This study investigated whether low- and high-proliferative soft tissue sarcomas can be differentiated using conventional imaging and radiomics features on MRI.

**Methods:**

In this retrospective study, soft tissue sarcomas were separated into two groups according to their proliferative activity: high-proliferative (Ki-67 ≥ 20%) and low-proliferative soft tissue sarcomas (Ki-67 < 20%). Several radiomics features, and various conventional imaging features on MRI like tumor heterogeneity, peritumoral edema, peritumoral contrast-enhancement, percentage of ill-defined tumor margins, Apparent Diffusion Coefficient (ADC) values, and area under the curve (AUC) in contrast dynamics were collected. These imaging features were independently compared with the two mentioned groups.

**Results:**

118 sarcoma cases were included in this study. Metastases were more prevalent in high-proliferative soft tissue sarcomas (*p* < 0.001), and time till metastasis negatively correlated with the Ki-67 proliferation index (k -0.43, *p* = 0.021). Several radiomics features representing intratumoral heterogeneity differed significantly between both groups, especially in T2-weighted (T2w) and contrast-enhanced T1-weighted (CE-T1w) sequences. Peritumoral contrast enhancement and edema were significantly more common in soft tissue sarcomas with a high Ki-67 index (*p* < 0.001). Tumor configuration, heterogeneity, and ill-defined margins were commonly seen in high-proliferative soft tissue sarcomas (*p* = 0.001–0.008). Diffusion restriction (ADC values) and contrast dynamics (AUC values) did not present significant differences between low- and high-proliferative soft tissue sarcomas.

**Conclusions:**

Several radiomics and conventional imaging features indicate a higher Ki-67 proliferation index in soft tissue sarcomas and can therefore be used to distinguish between low- and high-proliferative soft tissue sarcomas.

## Introduction

Soft tissue sarcomas (STS) are rare tumors of mesenchymal origin. Around 80 subtypes are recognized, leading to a heterogeneous composition of this tumor group. Incidence lies around 4–5/100.000/ year in Europe, with increasing mortality due to insufficient advancements in prevention, diagnosis, and treatment [1, 2]. Several studies have already investigated the association of MRI features with tumor grade, revealing that, for example, multilobulated/ polycyclic tumor shape, intratumoral heterogeneity, and peritumoral contrast enhancement, as well as peritumoral edema, are associated with high-grade soft tissue sarcoma [3–9]. However, tumor grading and other tumor features are relevant in the soft tissue sarcoma prognosis. A High Ki–67 level—usually classified as > 20%—represents an independent prognostic marker in soft tissue sarcomas, indicating early metastasis and decreased survival [10–14]. Ki-67 is an IgG1 class murine monoclonal antibody. The name derives from the city of Kiel, where it was found, and the antibody-producing clone in the 67th tissue culture plate. The Ki-67 antigen is associated with the nucleus of the cell (depending on the cell cycle phase in late G1 phase perinucleolar, in S phase homogeneously in karyoplasm, in G2 granular in the karyoplasm and pro- and metaphase perichromosomal) and is expressed in every part of the cell cycle except G0 phase [15]. The Ki-67 antigen is a non-histone nuclear and nucleolar protein encoded by the MKI-67 gene (chromosome 10q26.2) [16]. The antigen is expressed in two isoforms encoded by two splice variants [17]. The epitope for the original antibody, also called the ‘Ki-67 motif’, lies within a tandem repeat re-gion of the protein. Since the discovery of the Ki-67 antibody, further antibodies against the Ki-67 protein have been found, like MIB-1 [17]. Ki-67 levels in the cell cycle are controlled by mRNA transcription and protein degradation [17]. The function of the Ki-67 antigen is still not entirely clear. Some researchers proposed that the Ki-67 antigen functions as a surfactant, enabling chromosome motility and its interaction with the mitotic spindle and preventing chromosomes from collapsing into chromatin mass after nuclear envelope disassembly [18]. Other studies have claimed that Ki-67 plays a role in rRNA transcription and ribosome biogenesis [16]. Only a few studies have investigated the association of imaging features and proliferative activity as measured by the Ki-67 index. They found a significant association with diffusion restriction [19–22], an association with the evolvement of necrosis and heterogeneity in T2-weighted sequences (T2w) [23], and an association with peritumoral T2w hyperintensity [24]. Further information on the correlation of imaging features with the Ki-67 proliferation index might contribute valuable supplementary information to understanding soft tissue sarcoma tumor biology and radiologic reporting regarding prognosis and potential therapy planning. Therefore, this study aimed to investigate whether radiomics and conventional MRI imaging features can help distinguish soft tissue sarcomas with low- and high-proliferative activity indicated by the Ki67 index.

## Materials and methods

### Study design

The institutional review board approved this retrospective study, and all patients gave their verbal informed consent before examination. The local radiologic information system was screened for sarcoma patients with the first diagnosis between 2013 and 2023. Patients were included according to the following inclusion criteria: (1) diagnosis of soft tissue sarcoma, (2) available immunohistochemical data including Ki-67 proliferation index, and (3) available pretherapeutic MRI imaging. Exclusion criteria were (1) MRI imaging rich in artifacts, (2) uterine leiomyosarcomas, (3) gastrointestinal stromal tumors, or (4) primary intraosseous tumor location. If specific MRI sequences were lacking or had to be excluded due to artifacts, the other sequences were still included in the analysis. Baseline data include patients’ age, sex, tumor entity, presence of metastasis at the time of diagnosis or during follow-up in our hospital, and time until metastasis was documented. Furthermore, the Ki-67 index (mostly but not only from the in-house pathology lab, assessed according to lab standards) was documented. Sarcoma cases were divided into subgroups of patients with Ki-67 < 20% (low-proliferative) and ≥ 20% (high-proliferative), which was shown in previous studies to be prognostic relevant [10–14]. If the pathologist did not determine a precise percentage but a range of the Ki-67 index, the mean of this range was used for analysis.

### Conventional imaging features and Radiomics

Two readers with 4 and 8 years of experience performed the image analysis, blinded to immunohistochemical results. The findings were reached by consensus. Tumor configuration (categorized as (1) ovoid, (2) fusiform, (3) multilobulated/ polycyclic, or (4) streaky as proposed by previous studies [25, 26]), the extent of intratumoral necrosis, intratumoral hemorrhage and cystic degeneration, peritumoral edema, and peritumoral enhancement (categorized as (1) absent, (2) mild, (3) moderate or (4) extensive), the extent of intratumoral heterogeneity and volume (measured in two planes) were assessed. Furthermore, the average Apparent Diffusion Coefficient (ADC) was acquired from an ROI in the non-lipomatous tumor component and, for standardization, in healthy muscle. T1-weighted sequences (T1w), T2-weighted sequences (T2w), and contrast-enhanced T1-weighted sequences (CE-T1w) were imported in mint LesionTM software (v. 3.9.0, Mint Medical GmbH, Germany) and regions of interest (ROIs) drawn around the non-lipomatous tumor component. 213 radiomics features (71 in each sequence) were extracted, including first-order statistics and gray-level co-occurrence matrix (GLCM) features. The mean signal-time curve was extracted in syngo.via (v. 8.9, Siemens Healthcare GmbH, Germany) in patients with available contrast dynamics in an ROI of the tumor mass in the image slice with the maximum diameter and an ROI of a major artery. Time of arterial inflow was defined as an increase of at least 30% of the intensity in the major artery. The area under the curve (AUC) was calculated (by using the trapezoidal rule) of the contrast enhancement of the tumor without baseline T1w signal intensity for the time of 30 s and 60 s after arterial inflow and standardization set to the AUC of contrast-enhancement of the artery.

### Statistical analysis

Data was analyzed descriptively with mean and standard deviation for metric variables and total amount and percentage for categorical variables. Significance tests were performed using the Chi-squared and Fisher exact tests for categorical variables and Student’s t-test and ANOVA for metric variables. Furthermore, Pearson correlation was performed until metastasis and Ki-67 proliferation index.

## Results

### Baseline characteristics

A total of 118 cases were included in this study. Of the low-proliferative soft tissue sarcoma, 36 (66.67%) were male and 18 (33.33%) female, similar to the group of high-proliferative tumors where 39 (60.94%) patients were male and 25 (39.06%) female. Likewise, the mean age was similar in both groups, with 50.09 years (± 28.41) in low-proliferative soft tissue sarcoma compared to 55.14 years (± 28.41) in high-proliferative soft tissue sarcoma. The most common tumor entity was myxofibrosarcoma in both groups, with a proportion of 14.81% in low-proliferative tumors and 20.31% in high-proliferative tumors. Distribution of tumor localization was similar, with the most common site being the extremities in both groups (68.52% and 76.56%, respectively; see Table 1).

**Table 1:** Baseline results

		Low-proliferative (amount (percentage) / mean (SD))	High-proliferative (amount (percentage) / mean (SD))
Sex	Male	36 (66.67%)	39 (60.94%)
	Female	18 (33.33%)	25 (39.06%)
Age		50.09 (± 28.41)	55.14 (± 28.41)
Entity	Myxofibrosarcoma	8 (14.81%)	13 (20.31%)
	Pleomorphic sarcoma		9 (14.06%)
	Myxoid liposarcoma	7 (12.96%)	
	Synovial sarcoma	7 (12.96%)	4 (6.25%)
	Leiomyosarcoma	5 (9.26%)	5 (7.81%)
	Dedifferentiated liposarcoma	4 (7.41%)	6 (9.38%)
	Solitary fibrous tumor	5 (9.26%)	
	Pleomorphic liposarcoma		5 (7.81%)
	Rhabdomyosarcoma		4 (6.25%)
	MPNST	1 (1.85%)	2 (3.13%)
	Fibrosarcoma	3 (5.56%)	
	Myofibroblastic sarcoma/ Evans tumor	2 (3.70%)	1 (1.56%)
	Spindle cell sarcoma	2 (3.70%)	1 (1.56%)
	Dermatofibrosarcoma protuberans	3 (5.56%)	
	FDC-Sarcoma		1 (1.56%)
	Sarcoma NOS		6 (9,38%)
	Other STS	7 (12,96%)	7 (10,94%)
Localization	Extremity	37 (68.52%)	49 (76.56%)
	Abdomen	12 (22.22%)	11 (17.19%)
	Thorax	4 (7.41%)	2 (3.13%)
	Head / Neck	1 (1.85%)	2 (3.13%)

**Fig. 1:**
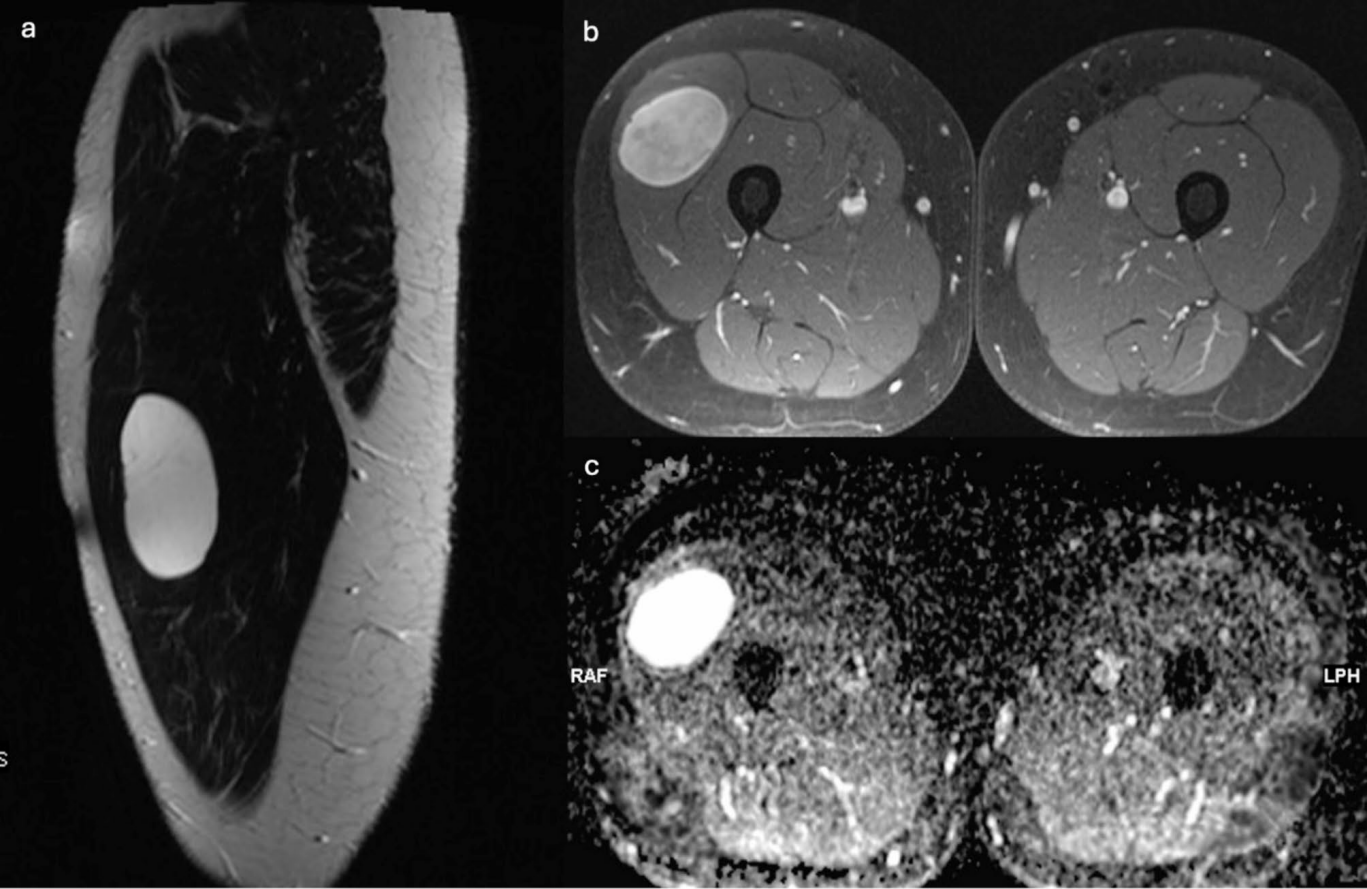
T2w homogenous myxoid liposarcoma of the right M. quadriceps femoris with high ADC, Ki-67 proliferation index 10%: (**a**) T2w TSE, (**b**) T1w TSE after contrast, (**c**) ADC map

**Fig. 2:**
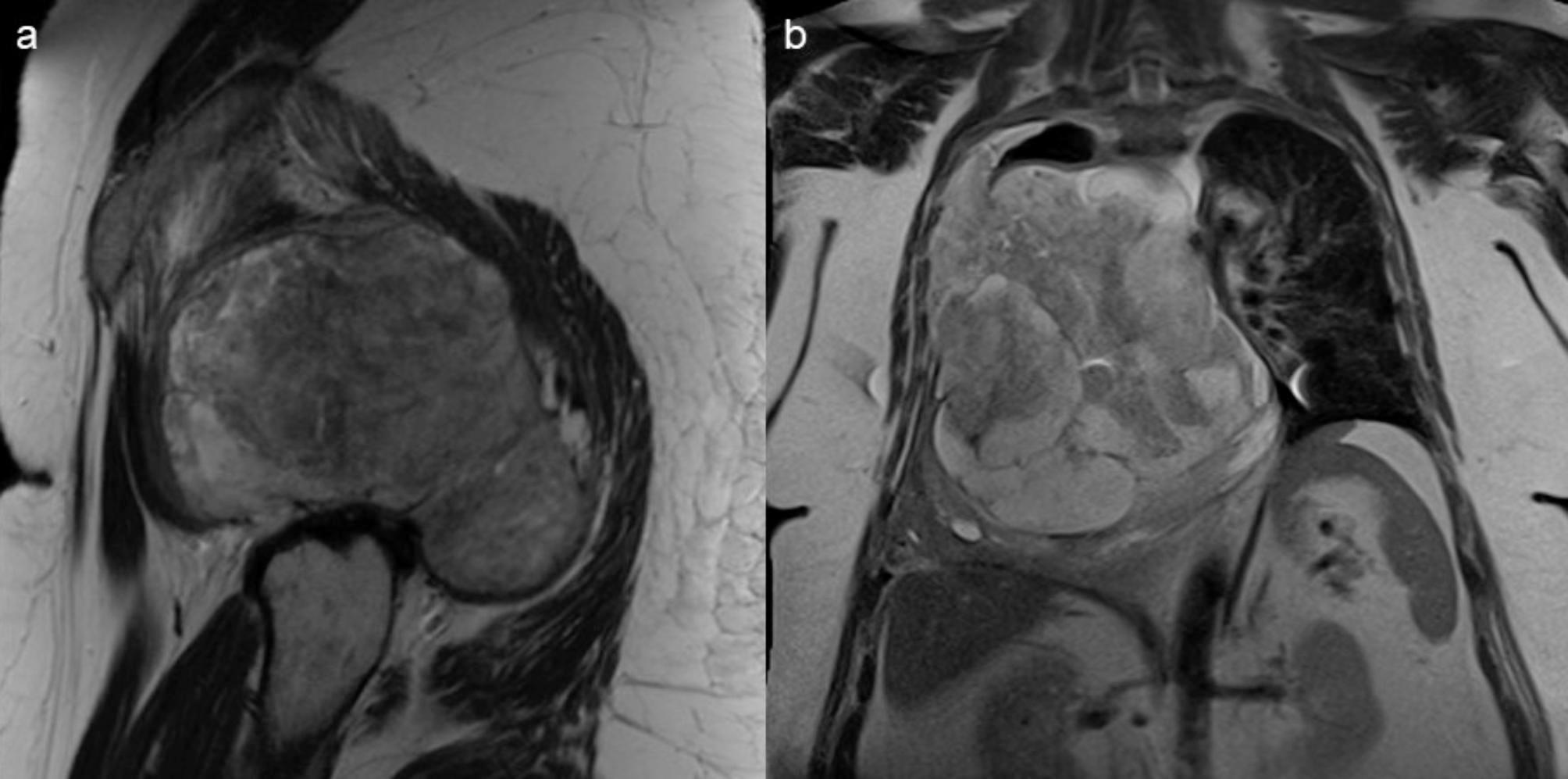
(**a**) T2w TSE of heterogenous gluteal undifferentiated small round cell sarcoma, Ki-67 index 85%, (**b**) T2w HASTE of heterogenous intimal sarcoma of the pulmonary artery, Ki-67 index 70%

Metastasis was more common in high-proliferative soft tissue sarcoma (*p* < 0.001). However, 16.98% of low-proliferative soft tissue sarcoma also presented metastasis. The mean time in months till metastasis was notably more extended for low-proliferative tumors with 27.00 (± 28.89) compared to 6.05 (± 6.67) in high-proliferative soft tissue sarcoma but without reaching a statistical significance level (*p* = 0.062). Still, time till metastasis did show a moderate correlation with the Ki67 proliferation index (k -0.43, *p* = 0.021). Furthermore, the high proliferative activity of soft tissue sarcoma, as measured by the Ki-67 proliferation index, was associated with tumor grade (see Table 2). Figures 1 and 2 show examples of different soft tissue sarcomas.

**Table 2:** Results from conventional imaging features and radiomics on MRI

Feature	Low-proliferative (amount (percentage)/ mean (SD))	High-proliferative (amount (percentage)/ mean (SD))	*p*-value
Volume (*n* = 117)	108.18 ml (± 67.67 ml)	124.89 ml (± 64.12 ml)	0.175
Configuration			**0.008**
* Ovoid*	26 (49.06%)	14 (21.88%)	
* Fusiform*	1 (1.89%)	1 (1.56%)	
* Multilobulated/ polycyclic*	25 (47.17%)	46 (71.88%)	
* Streaky*	1 (1.89%)	3 (4.69%)	
Subjective T2 Heterogeneity (*n* = 115)	49.09% (± 27.33%)	63.85% (± 23.58%)	**0.002**
Subjective CE-T1 Heterogeneity (*n* = 114)	48.00% (± 27.75%)	62.62% (± 23.62%)	**0.003**
Ill-defined Tumor margin T2 (*n* = 115)	12.91% (± 11.98%)	21.69% (± 11.98%)	**0.003**
Ill-defined Tumor margin CE-T1 (*n* = 114)	14.56% (± 15.21%)	24.72% (± 15.03%)	**0.001**
Extent intratumoral contrast enhancement (*n* = 114)	65.00% (± 26.51%)	59.92% (± 25.75%)	0.302
Tumor ADC mean (*n* = 63)	1366.41 (± 596.51)	1226.67 (342.71)	0.283
Tumor/Muscle mean ADC ratio (*n* = 63)	0.90 (± 0.44)	0.76 (± 0.23)	0.150
Extent necrosis (*n* = 115)	0.24 (± 0.23)	0.28 (± 0.24)	0.405
Extent cystic degeneration (*n* = 117)	4.57% (± 12.33%)	8.16% (± 16.11%)	0.184
Extent hemorrhage (*n* = 116)	6.09% (± 14.99%)	6.53% (± 12.21%)	0.862
Peritumoral edema (*n* = 115)			**< 0.001**
* Absent*	27 (50.94%)	8 (12.90%)	
* Mild*	12 (22.64%)	8 (12.90%)	
* Moderate*	9 (16.98%)	19 (30.65%)	
* Extensive*	5 (9.43%)	27 (43.55%)	
Peritumoral enhancement (*n* = 114)			**< 0.001**
* Absent*	28 (51.85%)	7 (11.67%)	
* Mild*	19 (35.19%)	21 (35.00%)	
* Moderate*	6 (11.11%)	18 (30.00%)	
* Extensive*	1 (1.85%)	14 (23.33%)	
Metastasis (*n* = 116)			**< 0.001**
* Not present*	44 (83.02%)	44 (69.84%)	
* Present*	9 (16.98%)	19 (30.16%)	
Months till metastasis	27.00 (± 28.89)	6.05 (± 6.67)	0.062
Tumor grade (*n* = 107)			**< 0.001**
* Grade 1*	27 (57.45%)	3 (5.36%)	
* Grade 2*	17 (36.17%)	17 (30.36%)	
* Grade 3*	3 (6.38%)	36 (64.29%)	
AUC contrast-enhancement tumor 30 s	887.92 (± 1084.35)	2046.27 (± 1758.13)	0.126
AUC contrast-enhancement tumor 60 s	3514.99 (± 4046.05)	6743.32 (± 4198.14)	0.105
AUC tumor / AUC artery 30 s	0.07 (0.07)	0.23 (± 0.26)	0.127
AUC tumor / AUC artery 60 s	0.14 (± 0.13)	0.33 (± 0.24)	0.069
T2 Histogram Variance	225519.18 (± 234748,69)	355012.44 (± 298473.18)	**0.014**
T2 Histogram Entropy	35231.64 (± 16265.00)	43040.29 (± 13474.03)	**0.008**
T2 Histogram Uniformity	0.09 (± 0.06)	0.06 (± 0.04)	**0.004**
T2 Histogram Median abs deviation	41980.73 (± 42790.56)	58039.77 (± 30717.15)	**0.026**
T2 Histogram Range	38.75 (± 29.08)	49.25 (± 23.72)	**0.041**
T2 Histogram Max	39.75 (± 29.08)	50.25 (± 23.72)	**0.041**
T2 GLCM Joint maximum	0.07 (± 0.07)	0.04 (± 0.04)	**0.01**
T2 GLCM Joint variance	230648.55 (± 238535.87)	350798.81 (± 291070.77)	**0.021**
T2 GLCM Joint entropy	59963.92 (± 26651.97)	70228.88 (± 25737.19)	**0.043**
T2 GLCM Angular second moment	0.03 (± 0.03)	0.01 (± 0.02)	**0.005**
CE-T1 Intensity Kurtosis	6581.73 (± 14221.78)	1604.05 (± 8140.14)	**0.029**
CE-T1 Intensity Variation	0.22 (± 0.10)	0.26 (± 0.09)	**0.008**
CE-T1 Intensity Quartile coefficient of dispersion	0.14 (± 0.09)	0.20 (± 0.08)	**0.002**

### Conventional imaging features

Tumor volume was slightly larger in high-proliferative tumors (108.18 ml ± 67.67 ml vs. 124.89 ml ± 64.12 ml) without reaching significance (*p* = 0.175). Significant differences between low-proliferative and high-proliferative sarcomas were observed in subjective T2w heterogeneity (49.10% vs. 63.85%, *p* = 0.002) and subjective CE-T1w heterogeneity (48.00% vs. 62.62%, *p* = 0.003). Likewise, tumor margin in T2w (12.91% ill-defined vs. 21.69% ill-defined, *p* = 0.003) and CE-T1w (14.56% vs. 24.72%, *p* = 0.001) was significantly less defined in high-proliferative tumors. Furthermore, a significant difference was found in the presence and extent of peritumoral enhancement: 88,33% of all high-proliferative sarcomas presented a peritumoral enhancement compared to 48,15% of all low-proliferative soft tissue sarcoma (*p* < 0.001). Likewise, there was a significant difference in the presence and extent of peritumoral edema: 87,1% of all high-proliferative soft tissue sarcoma showed peritumoral edema com-pared to 49,06% of the low-proliferative soft tissue sarcoma (*p* < 0.001, see Table 2). Additionally, ANOVA demonstrated a significant increase in the observed Ki-67 proliferation index with increasing peritumoral enhancement and peritumoral edema (both *p* < 0.001; see Table 3).

**Table 3:** Correlation of peritumoral contrast enhancement and edema with Ki67

Feature	Extent	Mean Ki67 (SD)	*p*-value
Peritumoral Enhancement	Absent	12.23% (± 15.05%)	< 0.001
	Mild	25.09% (± 20.27%)	
	Moderate	33.35% (± 21.06%)	
	Extensive	38.33% (± 21.06%)	
Peritumoral edema	Absent	14.56% (± 17.77%)	< 0.001
	Mild	17.53% (± 16.17%)	
	Moderate	28.57% (± 18.93%)	
	Extensive	38.73% (± 20.22%)	

The extent of necrosis was slightly higher in high-proliferative tumors (23.85% vs. 27.56%, *p* = 0.405) without reaching significance. There was almost no difference in cystic degeneration (4.57% vs. 8.12%, *p* = 0.184) and the extent of hemorrhage (6.09% vs. 6.53%, *p* = 0.862). The ADC was lower in highly proliferative tumors, but the difference was small and insignificant (1366.41 vs. 1226.67, *p* = 0.283). Similarly, after standardization with healthy muscle, the relative tumor ADC value showed no significant difference between both groups (0.8969 vs. 0.76008, *p* = 0.150). Twenty-two patients had available dynamic contrast-enhanced imaging (DCE). Of them, 7 were low-proliferative soft tissue sarcoma, and 15 were high-proliferative soft tissue sarcoma. The AUC after contrast enhancement was notably higher in the high-proliferative soft tissue sarcoma group with 2046.27 compared to 887.92 in the low-proliferative soft tissue sarcoma after 30 s and 6743.32 compared to 3514.99 after 60 s but without reaching significance (*p* = 0.126 and *p* = 0.105, respectively). Also, after standardization with the AUC of the artery, the same tendency was noted, especially after 60 s (*p* = 0.069); however, it still did not reach the significance level.

### Radiomics

Of the 71 analyzed radiomics features in each sequence, significant differences were found between low and high proliferating soft tissue sarcoma in T2w Intensity range (784.51 vs. 994.40, *p* = 0.042), T2w histogram variance (225519,18 vs. 355012,44, *p* = 0.014), T2w histogram entropy (35231.64 vs. 43040.29, *p* = 0.008), T2w histogram uniformity (0.091532 vs. 0.060336, *p* = 0.004), T2w histogram median absolute deviation (41980.73 vs. 58039.77, *p* = 0.026), T2w histogram range (38.75 vs. 49.25, *p* = 0.041), T2w GLCM Joint maximum (0.065392 vs. 0.037314, *p* = 0.010), T2w GLCM Joint variance (230648,54 vs. 350798,81, *p* = 0.021), T2w GLCM joint entropy (59963,92 vs. 70228,88, *p* = 0.043), T2w GLCM angular second moment (0.028447 vs. 0.013620, *p* = 0.005), CE-T1w intensity kurtosis (6581.73 vs. 1604.05, *p* = 0.029), CE-T1w intensity variation (0.215 vs. 0.264, *p* = 0.008) and CE-T1w intensity quartile coefficient dispersion (0.144053 vs. 0.196361, *p* = 0.002). The radiomics features that showed significant differences are listed in Table 2. Other radiomics features did show similar strong tendencies towards higher heterogeneity in high-proliferative soft tissue sarcoma like CE-T1w histogram uniformity (0.09 vs. 0.07, *p* = 0.126), T2w GLCM sum of variance (268621,95 vs. 360223.68, *p* = 0.068), CE-T1w histogram quartile coefficient of dispersion (0.22 vs. 0.26, *p* = 0.077) or T2w GLCM inverse difference (0.55 vs. 0.50, *p* = 0.051) but without reaching significance.

## Discussion

This study investigates whether radiomics and conventional MRI imaging features can help distinguish soft tissue sarcomas with low- and high-proliferative activity indicated by the Ki67 index.

### Prognostic relevance of Ki-67

We confirmed the prognostic importance of the Ki-67 proliferation index, as established in previous literature [10–14]. In our study, a high Ki-67 index (≥ 20%) was associated with nearly twice the rate of metastasis compared to a low Ki-67 index (< 20%) in soft tissue sarcoma patients and correlated with a shorter time to metastasis.

### Size, configuration, and peritumoral changes

High proliferative soft tissue sarcoma was slightly larger than soft tissue sarcoma with low Ki-67 expression. However, the difference was small, and both groups showed a substantial standard deviation in size, which might also be attributed to differences in time till diagnosis, depending, for example, on tumor location. This finding aligns with previous studies by Kershaw et al. and Yang et al., who likewise did not find a significant correlation of Ki-67 with pretreatment soft tissue sarcoma size [24, 27]. Soft tissue sarcoma configuration/ shape is an established feature to differentiate low-grade and high-grade soft tissue sarcoma [25, 26, 28–31]. Our study’s tumor configuration differed between low and high proliferative activity. The high proliferative activity might lead to more irregular tumor growth with a multilobulated/ polycyclic configuration. The tumor margin is significantly less defined in high-proliferative soft tissue sarcoma, with the most significant difference being CE-T1w. However, the standard deviation was relatively high for both groups and distinguishing between them in clinical routines might be difficult. The difference is so tiny that an earlier study by Yang et al. has not found a significant difference in tumor margin [24]. However, Yang et al. did show an association of Ki-67 with peritumoral fat-saturated T2w hyperintensity [24]. Furthermore, a previous study by Lee et al. found a notable difference in peritumoral enhancement that almost reached significance [19]. In our more extensive analysis, peritumoral enhancement, as well as peritumoral edema, are more common and more extensive in high-proliferative tumors, which might reflect immunological and proangiogenic changes in the peritumoral environment, which is recognized for other tumor entities and might also apply for soft tissue sarcoma [32].

### Tumor heterogeneity and radiomics

Intratumoral heterogeneity of soft tissue sarcoma was associated with higher tumor grades [3]. Regarding proliferative activity, contrary to the results of the few previous studies [19, 24], the semantic image reading and the analyzed radiomics features revealed significant differences in intratumoral heterogeneity between low- and high-proliferative soft tissue sarcoma. Predominantly, T2w radiomics features showed significant differences between low-proliferative and high-proliferative soft tissue sarcoma, indicating higher heterogeneity in tumors with high proliferation. In a previous study, Meyer et al. investigated the association of radiomics features and Ki-67 and found a significant association for T2w Entropy, Sum of averages, and kurtosis [33]. We confirmed the significant difference in T2w histogram entropy and T2w GLCM Joint entropy. At the same time, the GLCM sum of averages and histogram of kurtosis did not reach significant differences, although our patient collective was larger. However, similar tendencies for these features were observed in our study. In our study, additional T2-weighted radiomics features representing tumor heterogeneity showed significant differences in high-proliferative tumors in first-order statistics and GLCM. Specifically, these features included intensity range, histogram variance, histogram uniformity, histogram median absolute deviation, histogram range, GLCM joint variance, and GLCM angular second moment. Although the most significant differences were found in T2w and CE-T1w, some radiomics features showed significant differences in intratumoral heterogeneity in high-proliferative soft tissue sarcoma: intensity kurtosis, intensity variation, and intensity quartile dispersion coefficient. Additionally, several radiomics features showed strong tendencies without reaching significance. High T2-weighted signals with missing enhancement in contrast-enhanced T1-weighted images can indicate intratumoral necrosis. Therefore, the increased intratumoral heterogeneity observed in high-proliferative soft tissue sarcoma might be due to these inhomogeneous necrotic areas. Correspondingly, Fadli et al. demonstrated a significant association between Ki-67 levels, the development of necrosis, and T2-weighted heterogeneity in pre-therapeutic MRIs [23]. It is established that fast-growing tumors, in general, induce high levels of angiogenesis but outgrow their vascular supply with chronic hypoxia and nutrient deprivation and, in some areas, necrosis or hypoxia-adapted regions as a consequence [34–37]. Conversely, necrosis was shown not to hinder but to enforce cancer progression and to increase proliferation (for example, through tissue inhibitor of metalloproteinases-1 (TIMP-1) and GCN2-ATF4 pathway) measured by Ki-67 [38, 39]. Therefore, higher tumor heterogeneity in high-proliferative soft tissue sarcomas may partly result from heterogenous necrosis caused by hypoxia and nutrient deprivation.

### Apparent diffusion coefficient and contrast dynamics

Previous studies showed a negative correlation with ADC for Ki-67 in the murine model of rhabdomyosarcoma and human soft tissue sarcoma patients [19, 20, 22, 40, 41]. In contrast, Kershaw et al. did not find a correlation between diffusion restriction and pretreatment Ki-67 [27]. In our study, mean ADC was lower in high-proliferative tumors, as could be expected, as low ADC values represent hypercellularity [42]. However, the difference was small and insignificant, although our study population was more extensive than in previous studies. A possible explanation for this might be the necrotic changes in high-proliferative tumors. In earlier studies on other tumor entities, no correlation was found between Ki-67 and ADC, ascribed to reduced cellularity through hypoxic necrosis [43]. In our study, the mean and maximum in T2w were higher in high-proliferative tumors, and the mean and maximum intensity in CE-T1w were lower for high-proliferative tumors, which might correlate to necrosis. However, the differences did not reach statistical significance.

While for some tumor entities like gastric cancer, lymphoma, or multiple myeloma, an association of proliferative activity with angiogenesis was shown [44–46], there was no association with hepatocellular carcinoma [47]. Furthermore, it was shown in previous literature that dynamic contrast imaging reflects the properties of tumor angiogenesis [48–52]. In our study, high-proliferative tumors presented a more intense early contrast enhancement 30 and 60 s after arterial inflow, which might reflect hypervascularity due to angiogenesis. However, the difference did not reach the level of significance, probably because of the relatively small number of patients with available contrast dynamics due to the retrospective nature of this study. However, Lee et al. and Kershaw et al. have not found a significant association between Ki-67 and dynamic contrast imaging features [19, 27]. Further studies on this issue are lacking. A prospective larger study focusing on contrast dynamics, including ktrans, might help investigate the contrast behavior of soft tissue sarcoma further regarding their proliferative activity and might help evaluate our findings further.

### Perspectives

The strength of our study lies in the relatively large cohort of patients with this rare tumor entity, coupled with the integration of both semantic image analysis and radiomics features, as well as the combination of intratumoral and peritumoral imaging characteristics. Our findings suggest the potential for radiomics-based prognostic estimation in soft-tissue sarcomas, which could guide therapy decisions based on the proliferative activity in soft tissue sarcomas without needing biopsy.

### Limitations

Our study has several limitations. First, due to the rarity of soft tissue sarcoma, it is a retrospective study. Therefore, not all patients had available diffusion imaging and dynamic contrast-enhanced imaging, which influences the evaluation of statistical significance. Second, only the AUC of the contrast dynamics could be calculated due to the retrospective study design. Without previously defined imaging protocols, it was not possible to calculate DCE features like ktrans. Third, this is a single-center study. Further studies should evaluate these features in a multicenter approach.

## Conclusion

We showed that several radiomics features representing tumoral heterogeneity reflect a higher proliferation rate, as described by Ki-67. Also, several conventional imaging features such as tumoral heterogeneity and configuration, peritumoral contrast enhancement and edema, and ill-defined margins indicate high-proliferative soft tissue sarcomas and, therefore, reduced prognosis. By knowing about these selected features, classifying the proliferation rate of soft tissue sarcomas could be eased. Future studies should investigate radiomics and conventional imaging features on MRI for the same purpose in larger cohorts.

## Data Availability

The datasets used and/or analyzed during the current study are available from the corresponding author on reasonable request.

## Abbreviations

STS: Soft tissue sarcoma
T1w: T1-weighted
T2w: T2-weighted
CE: T1w-Contrast-enhanced T1-weighted
ROI: Region of interest
GLCM: Gray-level co-occurrence matrix
ADC: Apparent Diffusion Coefficient
DCE: Dynamic contrast-enhanced

## References

[1] Pizzato M, Collatuzzo G, Santucci C, Malvezzi M, Boffetta P, Comandone A, Levi F, La Vecchia C, Bertuccio P, Negri E. Mortality patterns of soft-tissue sarcomas worldwide up to 2018, with predictions for 2025. Eur J Cancer Prev. 2023;32(1):71–80. 36346699 10.1097/CEJ.0000000000000768

[2] Gronchi A, Miah AB, Dei Tos AP, Abecassis N, Bajpai J, Bauer S, Biagini R, Bielack S, Blay JY, Bolle S, et al. Soft tissue and visceral sarcomas: ESMO-EURACAN-GENTURIS Clinical Practice Guidelines for diagnosis, treatment and follow-up(☆). Ann Oncol. 2021;32(11):1348–65. 34303806 10.1016/j.annonc.2021.07.006

[3] Schmitz F, Sedaghat S. Inferring malignancy grade of soft tissue sarcomas from magnetic resonance imaging features: A systematic review. Eur J Radiol. 2024;177:111548. 38852328 10.1016/j.ejrad.2024.111548

[4] Sedaghat S, Sedaghat M, Krohn S, Jansen O, Freund K, Streitburger A, Reichardt B. Long-term diagnostic value of MRI in detecting recurrent aggressive fibromatosis at two multidisciplinary sarcoma centers. Eur J Radiol. 2021;134:109406. 33254066 10.1016/j.ejrad.2020.109406

[5] Sedaghat S, Schmitz F, Meschede J, Sedaghat M. Systematic analysis of post-treatment soft-tissue edema and seroma on MRI in 177 sarcoma patients. Surg Oncol. 2020;35:218–23. 32920505 10.1016/j.suronc.2020.08.023

[6] Boudabbous S, Hamard M, Saiji E, Gorican K, Poletti PA, Becker M, Neroladaki A. What morphological MRI features enable differentiation of low-grade from high-grade soft tissue sarcoma? BJR Open. 2022;4(1):20210081. 36105415 10.1259/bjro.20210081 PMC9459866

[7] Crombe A, Le Loarer F, Stoeckle E, Cousin S, Michot A, Italiano A, Buy X, Kind M. MRI assessment of surrounding tissues in soft-tissue sarcoma during neoadjuvant chemotherapy can help predicting response and prognosis. Eur J Radiol. 2018;109:178–87. 30527301 10.1016/j.ejrad.2018.11.004

[8] Crombe A, Marcellin PJ, Buy X, Stoeckle E, Brouste V, Italiano A, Le Loarer F, Kind M. Soft-Tissue Sarcomas: Assessment of MRI Features Correlating with Histologic Grade and Patient Outcome. Radiology. 2019;291(3):710–21. 30964422 10.1148/radiol.2019181659

[9] Tordjman M, Honore C, Crombe A, Bouhamama A, Feydy A, Dercle L, Haddag L, Bouche PA, Ngo C, Le Cesne A, et al. Prognostic factors of the synovial sarcoma of the extremities: imaging does matter. Eur Radiol. 2023;33(2):1162–73. 35980435 10.1007/s00330-022-09049-y

[10] Engellau J, Bendahl PO, Persson A, Domanski HA, Akerman M, Gustafson P, Alvegard TA, Nilbert M, Rydholm A. Improved prognostication in soft tissue sarcoma: independent information from vascular invasion, necrosis, growth pattern, and immunostaining using whole-tumor sections and tissue microarrays. Hum Pathol. 2005;36(9):994–1002. 16153463 10.1016/j.humpath.2005.07.008

[11] Sorbye SW, Kilvaer TK, Valkov A, Donnem T, Smeland E, Al-Shibli K, Bremnes RM, Busund LT. Prognostic impact of Jab1, p16, p21, p62, Ki67 and Skp2 in soft tissue sarcomas. PLoS ONE. 2012;7(10):e47068. 23071715 10.1371/journal.pone.0047068 PMC3465267

[12] Ueda T, Aozasa K, Tsujimoto M, Ohsawa M, Uchida A, Aoki Y, Ono K, Matsumoto K. Prognostic significance of Ki-67 reactivity in soft tissue sarcomas. Cancer. 1989;63(8):1607–11. 2647278 10.1002/1097-0142(19890415)63:8<1607::aid-cncr2820630827>3.0.co;2-1

[13] Yildirim S, Ciftdemir M, Ustabasioglu FE, Ustun F, Usta U. Evaluation of the factors affecting survival and local recurrence in thigh soft tissue sarcomas. Jt Dis Relat Surg. 2024;35(1):130–7. 38108174 10.52312/jdrs.2023.1289 PMC10746889

[14] Heslin MJ, Cordon-Cardo C, Lewis JJ, Woodruff JM, Brennan MF. Ki-67 detected by MIB-1 predicts distant metastasis and tumor mortality in primary, high grade extremity soft tissue sarcoma. Cancer. 1998;83(3):490–7. 9690542 10.1002/(sici)1097-0142(19980801)83:3<490::aid-cncr18>3.0.co;2-r

[15] Brown DC, Gatter KC. Monoclonal antibody Ki-67: its use in histopathology. Histopathology. 1990;17(6):489–503. 2076881 10.1111/j.1365-2559.1990.tb00788.x

[16] Menon SS, Guruvayoorappan C, Sakthivel KM, Rasmi RR. Ki-67 protein as a tumour proliferation marker. Clin Chim Acta. 2019;491:39–45. 30653951 10.1016/j.cca.2019.01.011

[17] Remnant L, Kochanova NY, Reid C, Cisneros-Soberanis F, Earnshaw WC. The intrinsically disorderly story of Ki-67. Open Biol. 2021;11(8):210120. 34375547 10.1098/rsob.210120 PMC8354752

[18] Cuylen S, Blaukopf C, Politi AZ, Muller-Reichert T, Neumann B, Poser I, Ellenberg J, Hyman AA, Gerlich DW. Ki-67 acts as a biological surfactant to disperse mitotic chromosomes. Nature. 2016;535(7611):308–12. 27362226 10.1038/nature18610 PMC4947524

[19] Lee JH, Yoon YC, Seo SW, Choi YL, Kim HS. Soft tissue sarcoma: DWI and DCE-MRI parameters correlate with Ki-67 labeling index. Eur Radiol. 2020;30(2):914–24. 31630234 10.1007/s00330-019-06445-9

[20] Zhang K, Dai Y, Liu Y, Tao J, Pan Z, Xie L, Wang S. Soft tissue sarcoma: IVIM and DKI parameters correlate with Ki-67 labeling index on direct comparison of MRI and histopathological slices. Eur Radiol. 2022;32(8):5659–68. 35278121 10.1007/s00330-022-08646-1

[21] Li X, Tao J, Zhu Y, Yin Z, Zhang Y, Wang S. Soft tissue sarcoma: intravoxel incoherent motion and diffusion kurtosis imaging parameters correlate with the histological grade and Ki-67 expression. Acta Radiol. 2023;64(4):1546–55. 36259287 10.1177/02841851221131931

[22] Fang S, Yang Y, Tao J, Yin Z, Liu Y, Duan Z, Liu W, Wang S. Intratumoral Heterogeneity of Fibrosarcoma Xenograft Models: Whole-Tumor Histogram Analysis of DWI and IVIM. Acad Radiol. 2023;30(10):2299–308. 36481126 10.1016/j.acra.2022.11.016

[23] Fadli D, Kind M, Michot A, Le Loarer F, Crombe A. Natural Changes in Radiological and Radiomics Features on MRIs of Soft-Tissue Sarcomas Naive of Treatment: Correlations With Histology and Patients’ Outcomes. J Magn Reson Imaging. 2022;56(1):77–96. 34939705 10.1002/jmri.28021

[24] Yang Y, Zhang L, Wang T, Jiang Z, Li Q, Wu Y, Cai Z, Chen X. MRI Fat-Saturated T2-Weighted Radiomics Model for Identifying the Ki-67 Index of Soft Tissue Sarcomas. J Magn Reson Imaging. 2023;58(2):534–45. 36326136 10.1002/jmri.28518

[25] Sedaghat S, Salehi Ravesh M, Sedaghat M, Both M, Jansen O. Configuration of soft-tissue sarcoma on MRI correlates with grade of malignancy. Radiol Oncol. 2021;55(2):158–63. 33600679 10.2478/raon-2021-0007 PMC8042815

[26] Sedaghat S, Salehi Ravesh M, Sedaghat M, Meschede J, Jansen O, Both M. Does the primary soft-tissue sarcoma configuration predict configuration of recurrent tumors on magnetic resonance imaging? Acta Radiol. 2022;63(5):642–51. 33853376 10.1177/02841851211008381

[27] Kershaw L, Forker L, Roberts D, Sanderson B, Shenjere P, Wylie J, Coyle C, Kochhar R, Manoharan P, Choudhury A. Feasibility of a multiparametric MRI protocol for imaging biomarkers associated with neoadjuvant radiotherapy for soft tissue sarcoma. BJR Open. 2021;3(1):20200061. 35707756 10.1259/bjro.20200061 PMC9185851

[28] Sedaghat S, Sedaghat M, Meschede J, Jansen O, Both M. Diagnostic value of MRI for detecting recurrent soft-tissue sarcoma in a long-term analysis at a multidisciplinary sarcoma center. BMC Cancer. 2021;21(1):398. 33849475 10.1186/s12885-021-08113-y PMC8042876

[29] Sedaghat S, Schmitz F, Grozinger M, Sedaghat M. Malignant peripheral nerve sheath tumours in magnetic resonance imaging: primary and recurrent tumour appearance, post-treatment changes, and metastases. Pol J Radiol. 2020;85:e196–201. 32419885 10.5114/pjr.2020.94687 PMC7218449

[30] Sedaghat S, Schmitz F, Sedaghat M, Nicolas V. Appearance of recurrent dermatofibrosarcoma protuberans in postoperative MRI follow-up. J Plast Reconstr Aesthet Surg. 2020;73(11):1960–5. 32952057 10.1016/j.bjps.2020.08.089

[31] Sedaghat S, Surov A, Krohn S, Sedaghat M, Reichardt B, Nicolas V. Configuration of Primary and Recurrent Aggressive Fibromatosis on Contrast-Enhanced MRI with an Evaluation of Potential Risk Factors for Recurrences in MRI Follow-Up. Rofo. 2020;192(5):448–57. 31622987 10.1055/a-1022-4546

[32] Zhang S, Regan K, Najera J, Grinstaff MW, Datta M, Nia HT. The peritumor microenvironment: physics and immunity. Trends Cancer. 2023;9(8):609–23. 37156677 10.1016/j.trecan.2023.04.004 PMC10523902

[33] Meyer HJ, Renatus K, Hohn AK, Hamerla G, Schopow N, Fakler J, Josten C, Surov A. Texture analysis parameters derived from T1-and T2-weighted magnetic resonance images can reflect Ki67 index in soft tissue sarcoma. Surg Oncol. 2019;30:92–7. 31500794 10.1016/j.suronc.2019.06.006

[34] Hemmerlein B, Kugler A, Ozisik R, Ringert RH, Radzun HJ, Thelen P. Vascular endothelial growth factor expression, angiogenesis, and necrosis in renal cell carcinomas. Virchows Arch. 2001;439(5):645–52. 11764385 10.1007/s004280100464

[35] Evans SM, Hahn SM, Magarelli DP, Koch CJ. Hypoxic heterogeneity in human tumors: EF5 binding, vasculature, necrosis, and proliferation. Am J Clin Oncol. 2001;24(5):467–72. 11586098 10.1097/00000421-200110000-00011

[36] Evans SM, Hahn SM, Magarelli DP, Zhang PJ, Jenkins WT, Fraker DL, Hsi RA, McKenna WG, Koch CJ. Hypoxia in human intraperitoneal and extremity sarcomas. Int J Radiat Oncol Biol Phys. 2001;49(2):587–96. 11173159 10.1016/s0360-3016(00)01494-2

[37] Zhou J, Schmid T, Schnitzer S, Brune B. Tumor hypoxia and cancer progression. Cancer Lett. 2006;237(1):10–21. 16002209 10.1016/j.canlet.2005.05.028

[38] Karsch-Bluman A, Feiglin A, Arbib E, Stern T, Shoval H, Schwob O, Berger M, Benny O. Tissue necrosis and its role in cancer progression. Oncogene. 2019;38(11):1920–35. 30390074 10.1038/s41388-018-0555-y

[39] Ye J, Kumanova M, Hart LS, Sloane K, Zhang H, De Panis DN, Bobrovnikova-Marjon E, Diehl JA, Ron D, Koumenis C. The GCN2-ATF4 pathway is critical for tumour cell survival and proliferation in response to nutrient deprivation. EMBO J. 2010;29(12):2082–96. 20473272 10.1038/emboj.2010.81 PMC2892366

[40] Yuan Y, Zeng D, Liu Y, Tao J, Zhang Y, Yang J, Lkhagvadorj T, Yin Z, Wang S. DWI and IVIM are predictors of Ki67 proliferation index: direct comparison of MRI images and pathological slices in a murine model of rhabdomyosarcoma. Eur Radiol. 2020;30(3):1334–41. 31705255 10.1007/s00330-019-06509-w

[41] Yang Y, Fang S, Tao J, Liu Y, Wang C, Yin Z, Chen B, Duan Z, Liu W, Wang S. Correlation of Apparent Diffusion Coefficient With Proliferation and Apoptotic Indexes in a Murine Model of Fibrosarcoma: Comparison of Four Methods for MRI Region of Interest Positioning. J Magn Reson Imaging. 2023;57(5):1406–13. 35864603 10.1002/jmri.28371

[42] Schnapauff D, Zeile M, Niederhagen MB, Fleige B, Tunn PU, Hamm B, Dudeck O. Diffusion-weighted echo-planar magnetic resonance imaging for the assessment of tumor cellularity in patients with soft-tissue sarcomas. J Magn Reson Imaging. 2009;29(6):1355–9. 19472392 10.1002/jmri.21755

[43] Kim SY, Kim EK, Moon HJ, Yoon JH, Koo JS, Kim SG, Kim MJ. Association among T2 signal intensity, necrosis, ADC and Ki-67 in estrogen receptor-positive and HER2-negative invasive ductal carcinoma. Magn Reson Imaging. 2018;54:176–82. 30172938 10.1016/j.mri.2018.08.017 PMC7383359

[44] Patruno R, Zizzo N, Zito AF, Catalano V, Valerio P, Pellecchia V, D’Errico E, Mazzone F, Ribatti D, Ranieri G. Microvascular density and endothelial area correlate with Ki-67 proliferative rate in the canine non-Hodgkin’s lymphoma spontaneous model. Leuk Lymphoma. 2006;47(6):1138–43. 16840207 10.1080/10428190600565859

[45] Alexandrakis MG, Passam FH, Dambaki C, Pappa CA, Stathopoulos EN. The relation between bone marrow angiogenesis and the proliferation index Ki-67 in multiple myeloma. J Clin Pathol. 2004;57(8):856–60. 15280408 10.1136/jcp.2003.013110 PMC1770397

[46] Kikuyama S, Inada T, Shimizu K, Miyakita M. Thymidine phosphorylase expression in gastric cancer in association with proliferative activity and angiogenesis. Anticancer Res. 2000;20(3b):2081–6. 10928156

[47] Kitamura K, Hatano E, Higashi T, Narita M, Seo S, Nakamoto Y, Yamanaka K, Nagata H, Taura K, Yasuchika K, et al. Proliferative activity in hepatocellular carcinoma is closely correlated with glucose metabolism but not angiogenesis. J Hepatol. 2011;55(4):846–57. 21334407 10.1016/j.jhep.2011.01.038

[48] Yuan SJ, Qiao TK, Qiang JW, Cai SQ, Li RK. The value of DCE-MRI in assessing histopathological and molecular biological features in induced rat epithelial ovarian carcinomas. J Ovarian Res. 2017;10(1):65. 28950890 10.1186/s13048-017-0362-z PMC5615469

[49] Chen J, Chen C, Xia C, Huang Z, Zuo P, Stemmer A, Song B. Quantitative free-breathing dynamic contrast-enhanced MRI in hepatocellular carcinoma using gadoxetic acid: correlations with Ki67 proliferation status, histological grades, and microvascular density. Abdom Radiol (NY). 2018;43(6):1393–403. 28939963 10.1007/s00261-017-1320-3

[50] Kim E, Kim J, Maelandsmo GM, Johansen B, Moestue SA. Anti-angiogenic therapy affects the relationship between tumor vascular structure and function: A correlation study between micro-computed tomography angiography and dynamic contrast enhanced MRI. Magn Reson Med. 2017;78(4):1513–22. 27888545 10.1002/mrm.26547

[51] Xiao J, Rahbar H, Hippe DS, Rendi MH, Parker EU, Shekar N, Hirano M, Cheung KJ, Partridge SC. Dynamic contrast-enhanced breast MRI features correlate with invasive breast cancer angiogenesis. NPJ Breast Cancer. 2021;7(1):42. 33863924 10.1038/s41523-021-00247-3 PMC8052427

[52] Chen J, Qian T, Zhang H, Wei C, Meng F, Yin H. Combining dynamic contrast enhanced magnetic resonance imaging and microvessel density to assess the angiogenesis after PEI in a rabbit VX2 liver tumor model. Magn Reson Imaging. 2016;34(2):177–82. 26518059 10.1016/j.mri.2015.10.013

